# A Case Report of Facial Swelling and Crepitus Following a Dental Procedure

**DOI:** 10.21980/J83W8H

**Published:** 2025-07-31

**Authors:** Shady Mikhail, George Mina, Alisa Wray, Danielle Matonis

**Affiliations:** * University of California, Los Angeles Medical Center, Department of Internal Medicine, Los Angeles, CA; ^ University of California, Los Angeles Medical Center, Department of Anesthesia, Los Angeles, CA; † University of California, Irvine Medical Center, Department of Emergency Medicine, Orange, CA

## Abstract

Subcutaneous emphysema (SE) is a rare but significant complication following dental procedures characterized by the presence of air in the subcutaneous tissue. This case report presents a 67-year-old male who developed right-sided facial swelling after tooth extraction, a procedure executed with high-power dental tools. Clinical findings included facial swelling and crepitus with no lip involvement, differentiating SE from an allergic reaction and hematoma. Diagnostic imaging through computed tomography (CT) and chest X-ray confirmed SE, showing diffuse subcutaneous air in facial and neck soft tissues. Initial management in the emergency department (ED) involved administration of antihistamines, corticosteroids, antibiotics, and otolaryngology (ENT) consultation with close monitoring for airway compromise. This case underscores the importance of including SE in differential diagnoses for post-dental procedure swelling, the effectiveness of CT imaging in SE identification, and the importance of early detection and treatment to prevent severe complications like respiratory and cardiac issues.

**Topics:**

Subcutaneous emphysema; facial swelling; computed tomography (CT); dental procedure; emergency medicine.

**Figure:**
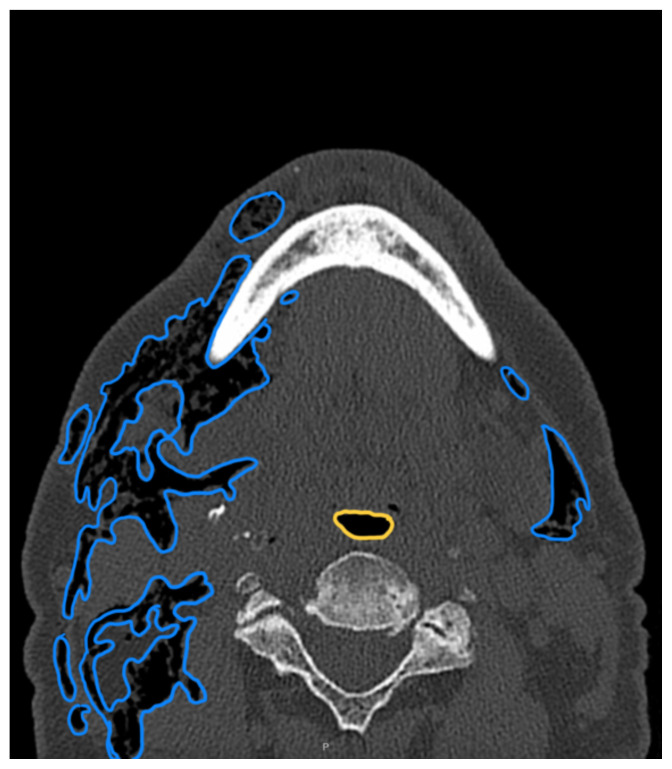


**Figure:**
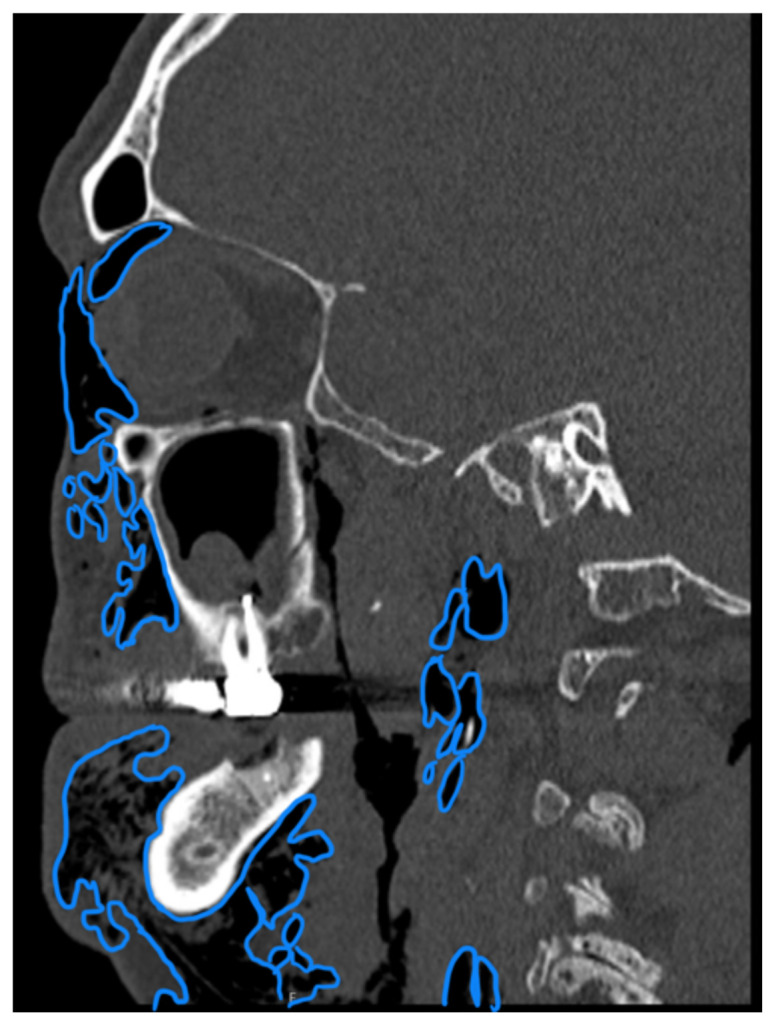


**Figure:**
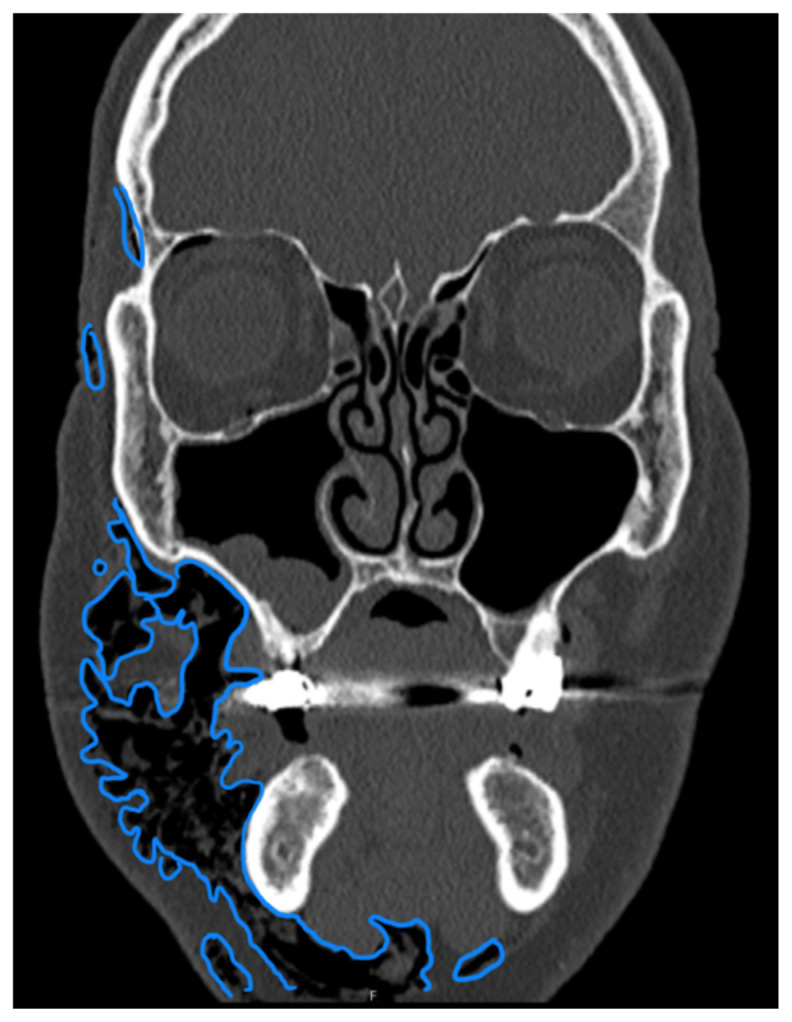


**Figure:**
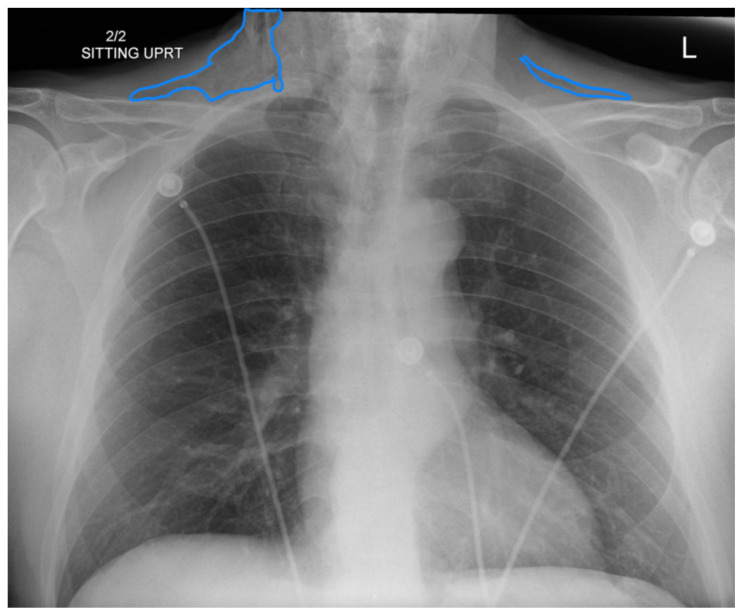


**Figure:**
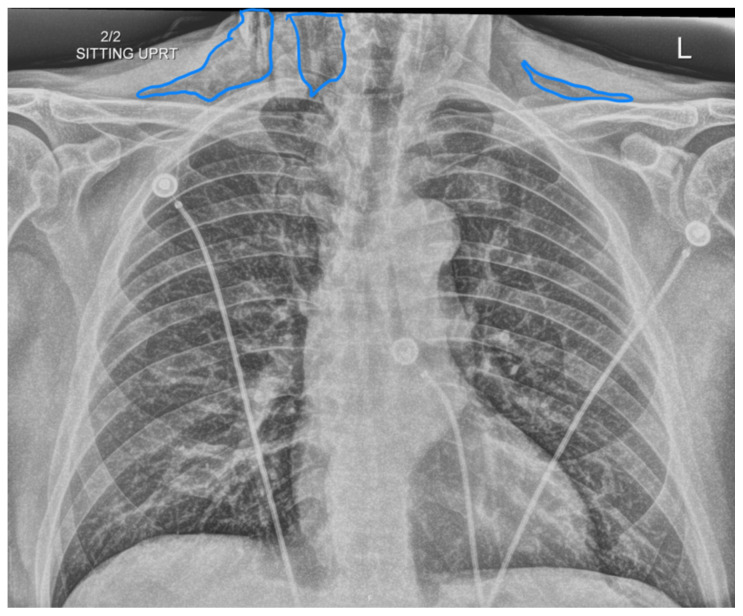


## Brief introduction

Subcutaneous emphysema (SE) is defined as the presence of air under the skin and in between soft tissue. A rare complication, SE is associated with dental procedures, though most cases of SE are preceded by dental procedures—approximately 54% of SE cases according to a systematic review spanning 23 years.^1^, ^2^ While infection and hematoma are considered more common post-operative complications, SE remains an important differential diagnosis.^3^ Although SE is often self-limiting, it can lead to life-threatening consequences, such as airway collapse and death.^4^ As a result, some important physical exam findings that may be present include swelling of the chest, neck, or face with lip sparing and crepitus on palpation.^5^ It is important to consider SE in the differential because it is often misidentified as an allergic reaction due to difficulty breathing from the swelling, but allergic reactions often involve lip swelling.^5^ The gold standard for diagnosing SE is X-ray and computerized tomography (CT), with CT being more sensitive to potentially identifying the source of the air.^6^ Here, we present a case of subcutaneous emphysema following a dental extraction and discuss the general management of SE.

## Presenting concerns and clinical findings*:*

A 67-year-old male with no reported past medical history presented with right-sided facial swelling one hour after a tooth extraction. The extraction was conducted with local anesthesia and no sedation. He was at the dentist to remove two anchor teeth for a 20-year-old bridge. Given the nature of the procedure and common practice for molar extractions, especially one with a high degree of decay, it is likely that a high-power air-pressure tool was used. He reported that the swelling started when the second tooth was being extracted. The swelling occurred suddenly and was reduced when he applied ice to the swelling. His dentist sent him to the ED for evaluation. He had tooth pain before the extraction but denied pain on arrival to the ED. He also denied recent fevers, vision changes, shortness of breath, rashes, itching, or vision changes.

The patient was afebrile with a temperature of 36.8^o^C, heart rate of 67 beats per minute, blood pressure of 119/72 mmHg, respiratory rate of 18 breaths per minute, and oxygen saturation of 96% on room air. The physical exam was notable for right cheek swelling without tenderness or erythema, clot at the extraction site, and sutures in place. No bleeding on the right inferior gumline was noted. The right inferior molars were surgically removed. There was notable periorbital swelling, most significantly on the right inferior eyelid that extended to the right cheek and neck with crepitus. There was no stridor or respiratory distress at rest.

## Significant findings

Complete blood count was significant only for elevated white blood cells to 14300 cells/μL and elevated absolute neutrophil count to 13100 cells/μL. The basic metabolic panel and coagulation tests were unremarkable. Given the physical exam findings of crepitus on the right neck up to the right lower eyelid, a maxillofacial CT scan without contrast was performed. It revealed diffuse subcutaneous air within the soft tissues of the face and neck and free air within the pre-septal soft tissue of the right eye, appearing as hyperlucent (dark) areas on CT within the soft tissue planes (blue outline). It showed no evidence of post-septal free air. A single-view chest X-ray was also performed and was unremarkable except for incompletely imaged soft tissue gas in the right lower neck (blue outline). On flexible fiberoptic laryngoscopy performed by ENT, the oropharynx appeared diffusely edematous and narrowed.

## Patient course

The diagnosis was determined to be subcutaneous emphysema in the setting of recent usage of high-power dental tools. Other potential causes of the patient’s swelling, such as postoperative hematoma and infection, were also considered. However, the rapid onset of symptoms—within one hour of the dental procedure—along with the absence of fever, erythema, or purulent drainage, made infection less likely. The elevated white blood cell count was interpreted as reactive leukocytosis. Hematoma was also entertained in the differential diagnosis, but the presence of crepitus on exam, absence of ecchymosis or fluctuance, and CT imaging that confirmed subcutaneous air without fluid collection helped rule out a hematoma. The patient was initially treated in the ED with one dose of diphenhydramine 25 mg and prednisone 60 mg in case there was a component of allergic reaction, and otolaryngology was consulted. They recommended one dose of dexamethasone 10mg, a seven-day course of amoxicillin-clavulanate 875-125 mg, and continued observation in ED in the event of airway compromise given the degree of airway edema seen on their scope. The patient remained in the emergency department under observation for approximately eight hours, during which he was reassessed and counseled about the risks of premature discharge, including potential airway compromise. Despite this, the patient refused to stay longer and decided to leave against medical advice. Strict return precautions were reviewed, and he was discharged with a prescription of amoxicillin-clavulanate 875-125 mg twice daily. Unfortunately, the patient was subsequently lost to follow-up. As a result, we were unable to assess progression or resolution of his subcutaneous emphysema beyond the initial encounter. This underscores the importance of patient education on return precautions and the need for close monitoring in cases with potential airway compromise.

## Discussion

Subcutaneous emphysema (SE), characterized by the presence of air in the subcutaneous layer of the skin, is a rare complication following dental procedures. In this scenario, SE typically causes swelling and pain localized to the face, neck, or chest, depending on the site and extent of air infiltration. Regardless of the etiology, SE presents with a palpable crackling sensation on physical exam, and in rare occasions, dyspnea, respiratory distress, air embolism, or pneumothorax.^7^,^8^ Rapid identification of these symptoms is crucial for timely intervention. The true incidence of SE has been difficult to establish due to underreporting in the literature and variable clinical presentations.^2^,^8^ The incidence of SE following dental procedures has also been reported variably in the literature. However, a systematic review spanning 23 years reports that the majority, around 54%, of SE cases were attributed to dental work.^2^ Traumatic intubation, tracheostomy, esophageal perforation, and central venous access procedures can all also lead to SE.^9^

Subcutaneous emphysema in the setting of dental work commonly happens following tooth extractions, restorative treatments, endodontic treatments, dental restorative treatments, and subgingival curettage.^8^ Generally, the use of high-pressure tools with air- or water-driven headpieces is the primary cause for SE and more likely to lead to higher degree of air infiltration and high likelihood of subsequent complications; moreover, procedures involving the posterior or mandibular teeth have been linked to higher rates of complications because those anatomical locations allow easier subcutaneous air infiltration.^2^ Individuals with compromised oral health, such as those with periodontal disease or poorly attached gingiva, have increased risk of SE following dental work.^10^

SE is generally a benign and self-resolving condition that rarely leads to serious complications. However, in some cases, extensive emphysema can lead to disfigurement, pneumothorax, airway compromise, respiratory failure, and even death.^4^,^11^ Additionally, if SE extends to the level of the thoracic outlet, not only can it compress the trachea, but it can also lead to compression of the great vessels, compromising venous return and cerebral blood flow.^12^

When SE is suspected, X-ray or CT imaging can be used to confirm, with CT being a superior tool to radiography.^6^ On X-ray, SE appears as radiolucent areas; on CT, it appears as dark spots.^6^ While the literature does not discuss the sensitivity of CT in diagnosing dental-work-induced SE, it was reported that CT has 85% sensitivity for diagnosing SE due to tracheal rupture.^13^ While SE can sometimes be diagnosed clinically—especially with mild symptoms and absence of systemic or airway concerns—imaging remains a valuable tool to confirm diagnosis and the extent of emphysema. In our case, imaging was warranted due to the rapid onset of significant facial swelling near the orbit and neck and the need to exclude other causes such as infection or angioedema. Similarly, ENT consultation was pursued because of the proximity of swelling to the airway and evidence of edema and crepitus on physical exam. Therefore, imaging and/or consultation are not universally required for all cases of SE, but can be useful tools in certain cases.

General initial treatment approaches involve conservative management, including antibiotics to prevent infection and close monitoring for potential respiratory complications. While cases of SE following dental procedures usually resolve without complications, rare cases of secondary infections such as cellulitis and abscess formation have been reported in the literature.^8^ The overall incidence of secondary infections remains largely unreported due to the rarity of the complication. While uncommon, respiratory complications such as pneumothorax, pneumomediastinum, and upper airway obstruction can occur as a sequelae of SE.^7^ In one case report, the emphysema was so extensive, the patient was taken for surgical exploration to assess for necrotizing infection.^14^ In our case, the rapid onset of symptoms, absence of systemic infection signs, and findings on imaging supported the diagnosis of SE over a necrotizing infection.

Unfortunately, there is no consensus on the specific treatment approach for SE. Most of the literature suggests that it is beneficial to start corticosteroids and antibiotics with close monitoring of the symptoms.^15^ This treatment approach can be beneficial for SE resulting from dental procedures. Depending on the mechanism of how SE was caused, the treatment plan can be different.^16^ For example, if a patient sustains an injury resulting in SE during endotracheal intubation, tracheostomy is indicated in this case.^16^ In the case of the injury occurring while the patient is mechanically ventilated, it is recommended to reduce air trapping which will prevent bronchospasm and the progression of SE.^17^

One limitation of this case is the lack of follow-up data because the patient elected to leave the emergency department against medical advice and did not return for further evaluation. This restricted our ability to track the progression or resolution of his subcutaneous emphysema. Nevertheless, this outcome reflects a common challenge in emergency settings—namely, the unpredictability of patient adherence—and underscores the importance of emphasizing return precautions and close outpatient monitoring in cases where airway compromise is a potential risk.

In this case, we highlighted the importance of keeping SE and its complications on the differential for facial swelling, especially after dental procedures such as tooth extractions. Although our patient left against medical advice after getting treated, and his SE was self-limiting, it is not always the case. It is crucial for SE to be diagnosed early in the disease course to avoid serious complications.

## Lessons for Clinicians

- Key physical exam findings can help differentiate between SE and an allergic reaction.
- It is crucial to monitor patients with facial SE for airway compromise.
- While rare, SE can result in complications such as respiratory compensation and cardiac arrest.

## Supplementary Information




















