# Supplementary figures and images for: A Case Report of Facial Swelling and Crepitus Following a Dental Procedure
Source: J Educ Teach Emerg Med. 2025 Jul 31;10(3):V8–V12. doi: 10.21980/J83W8H (PMC12321002; doi:10.21980/J83W8H)

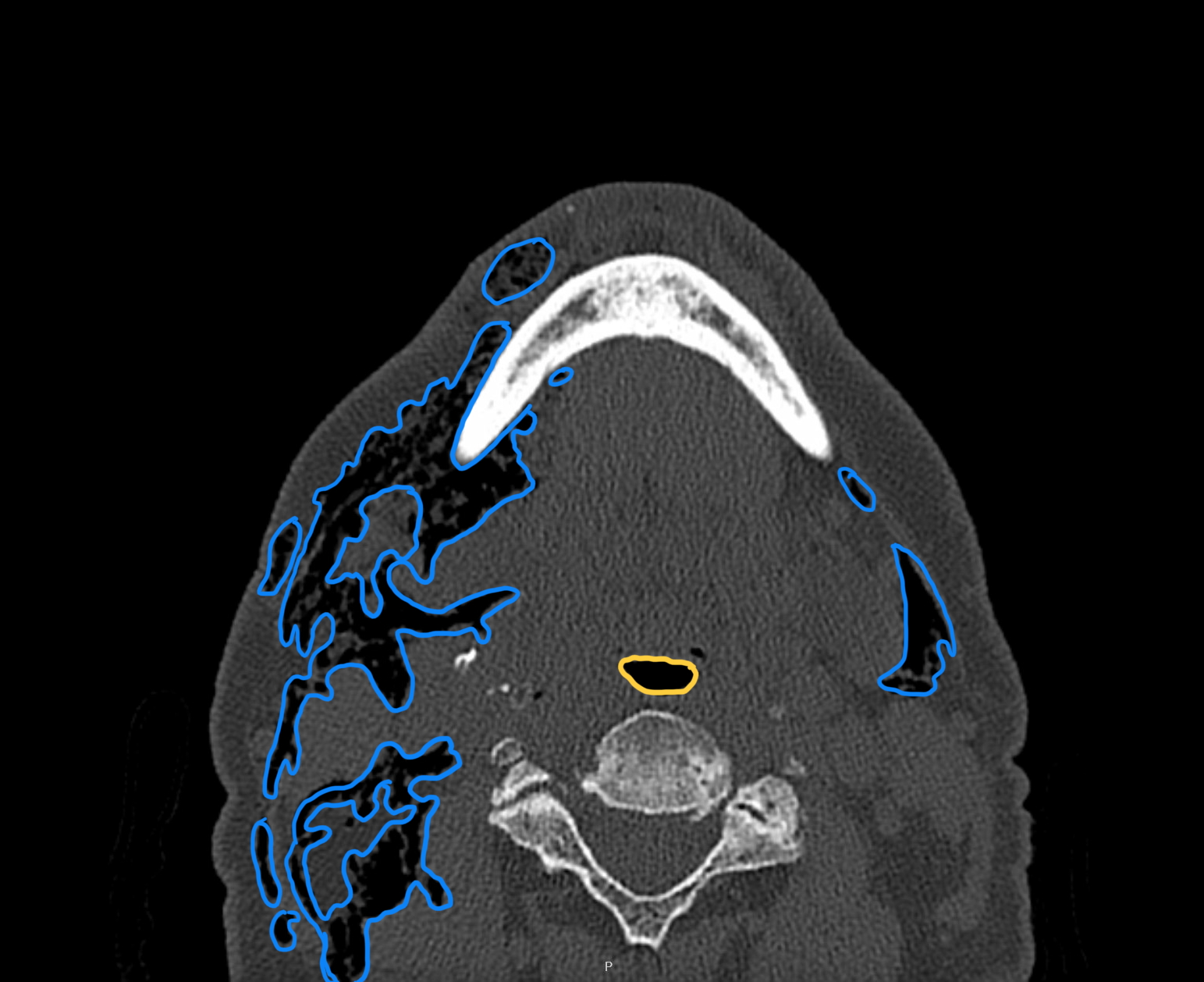

Supplement: Supplementary file 1 [file 10-3-V8-Supp1.jpeg]

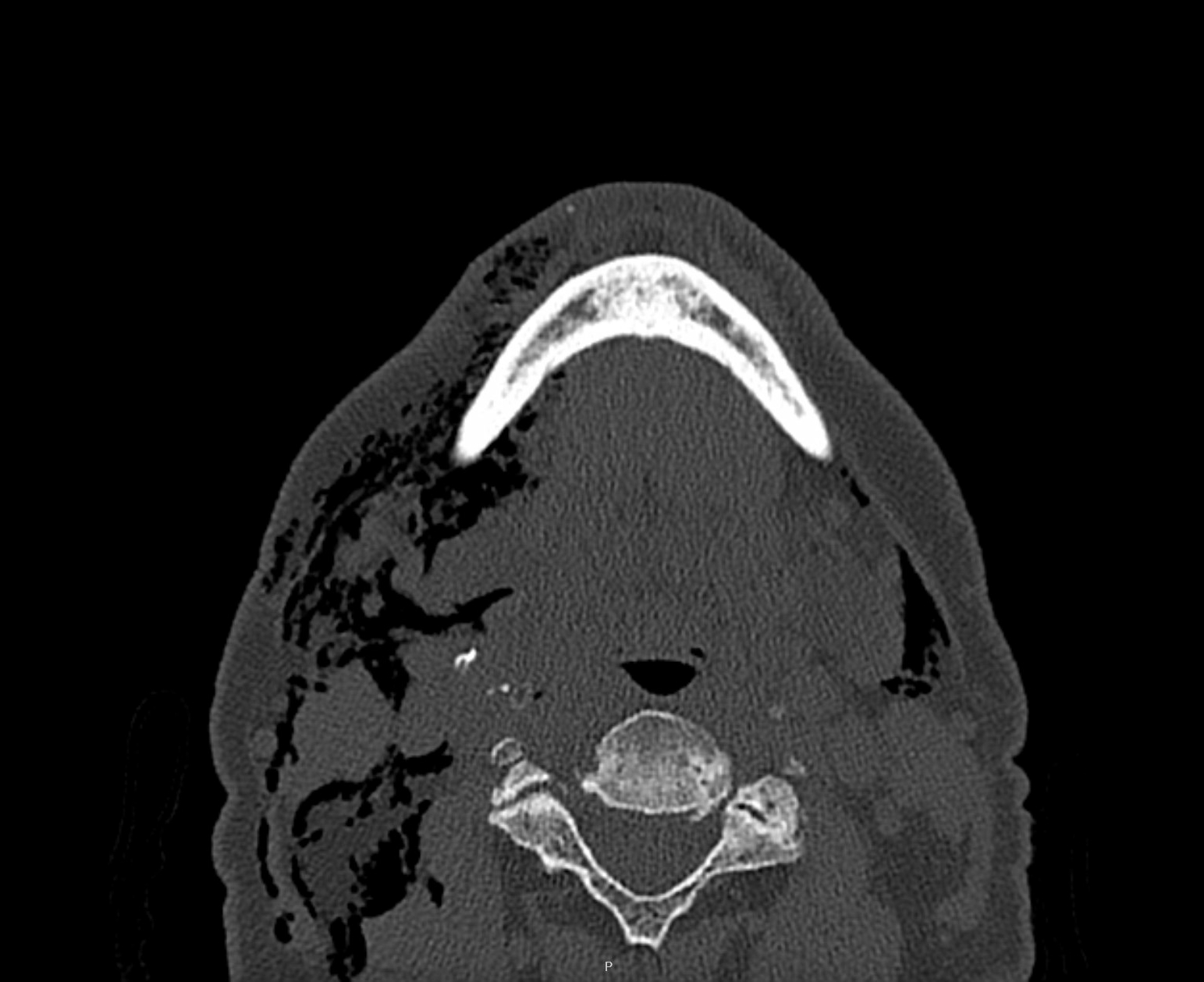

Supplement: Supplementary file 2 [file 10-3-V8-Supp2.jpeg]

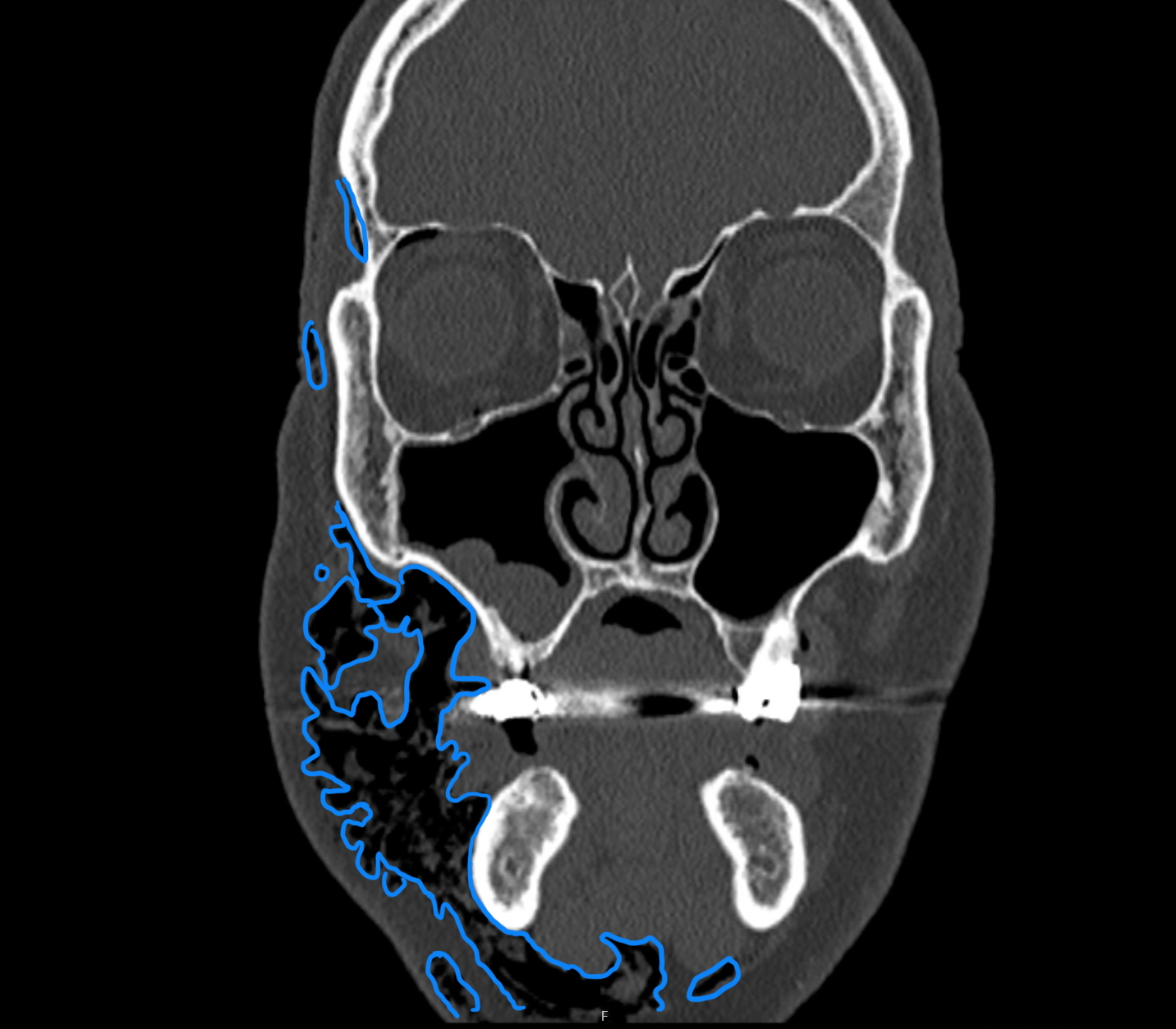

Supplement: Supplementary file 3 [file 10-3-V8-Supp3.jpeg]

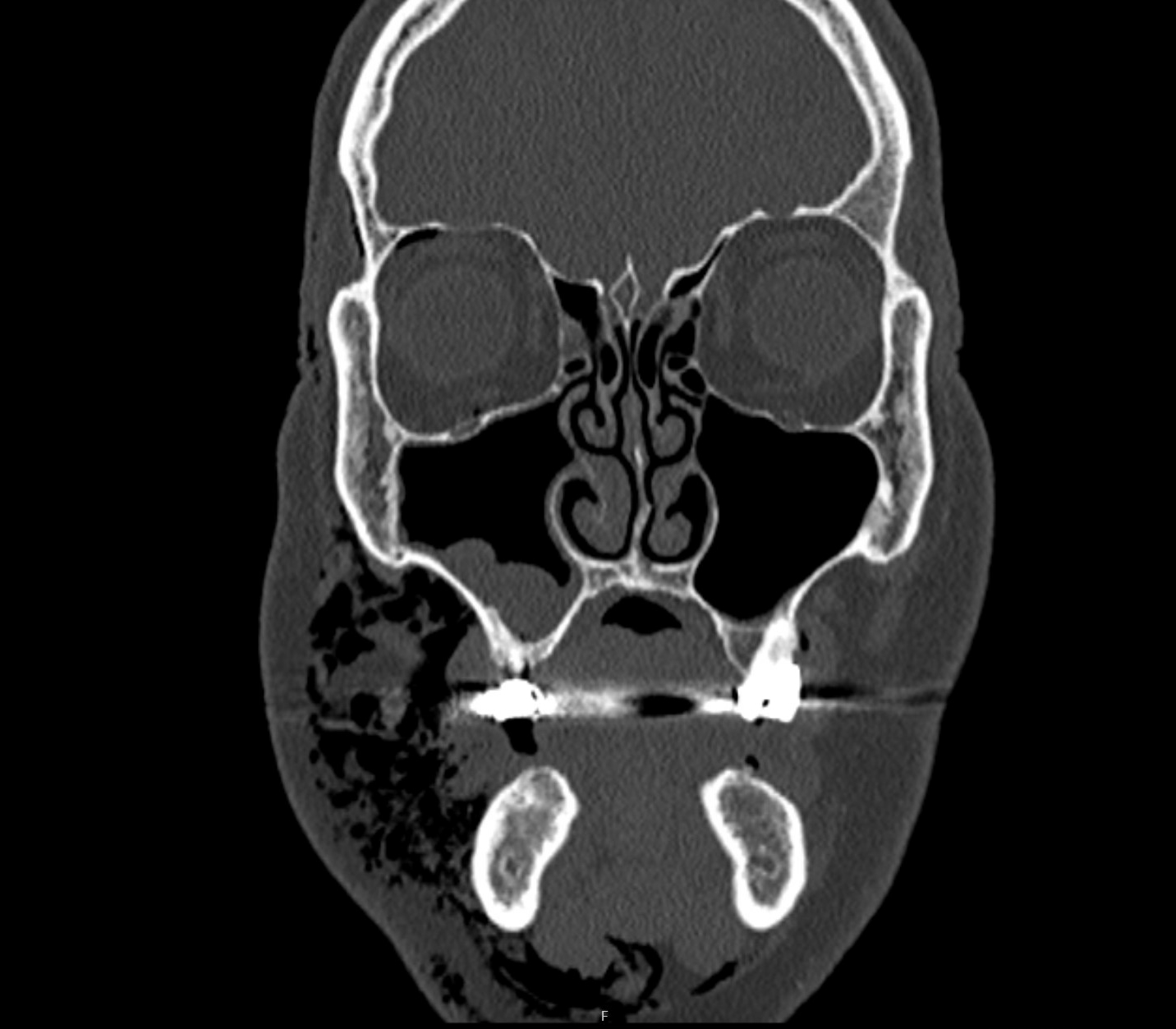

Supplement: Supplementary file 4 [file 10-3-V8-Supp4.jpeg]

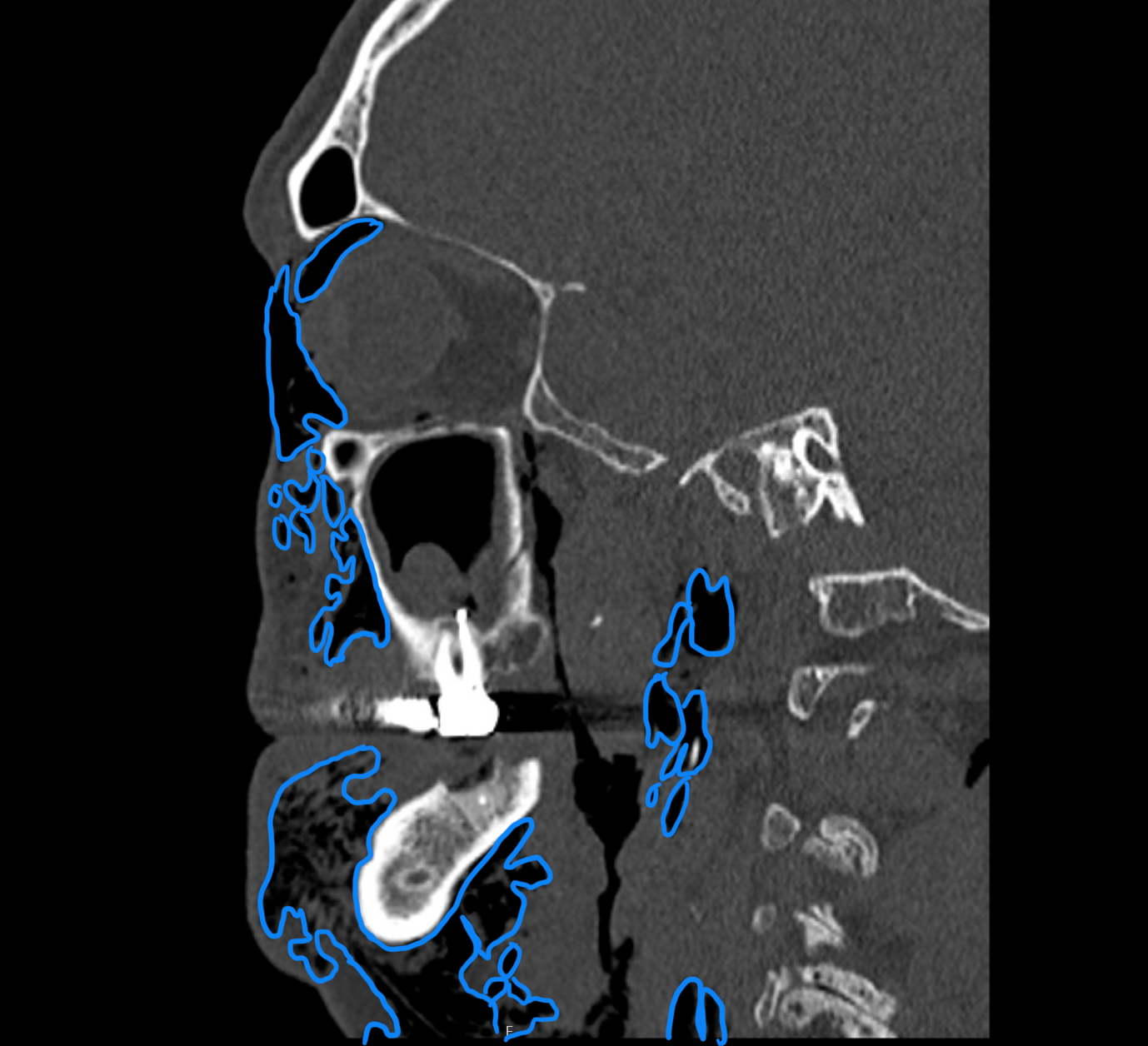

Supplement: Supplementary file 5 [file 10-3-V8-Supp5.jpeg]

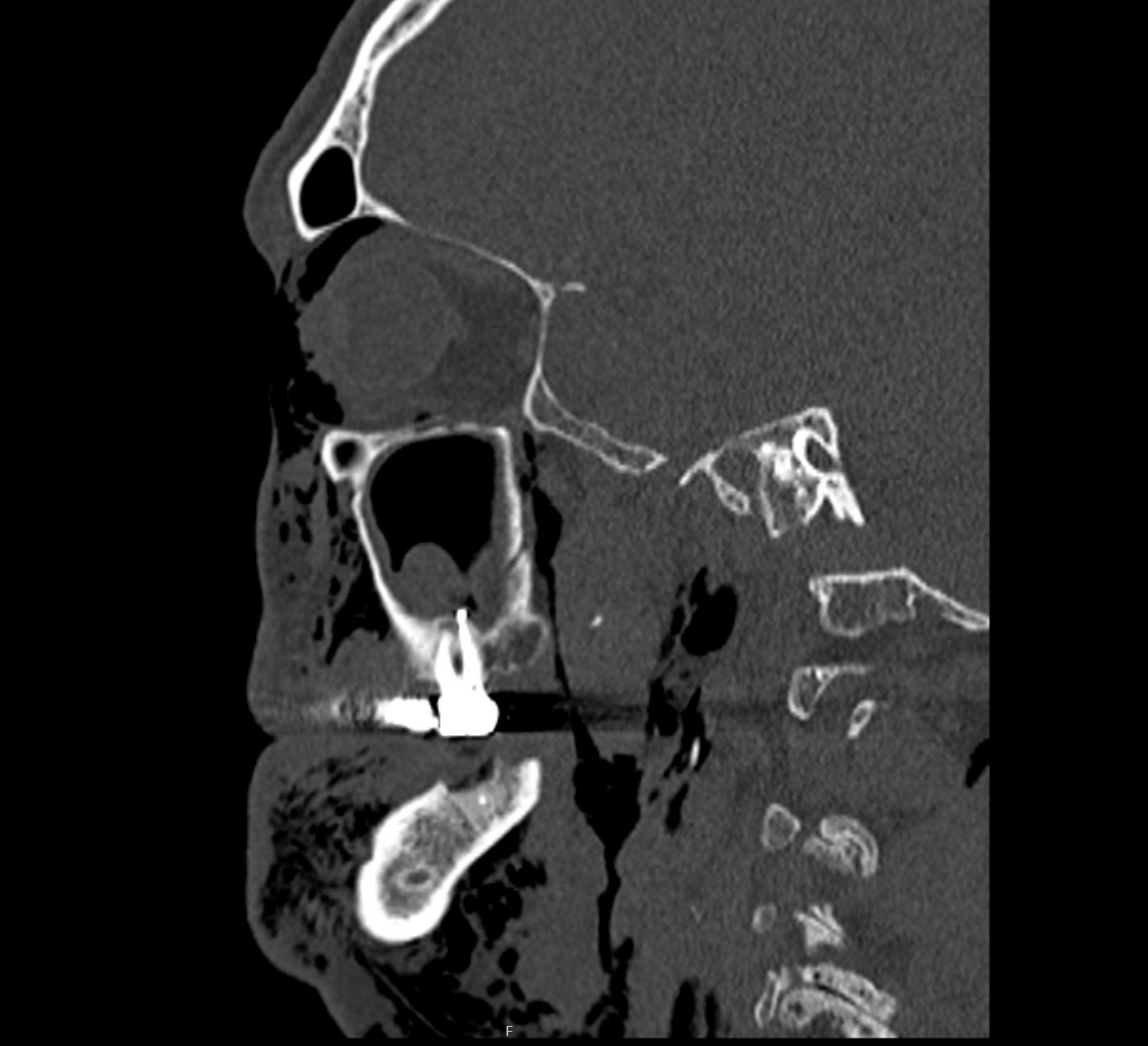

Supplement: Supplementary file 6 [file 10-3-V8-Supp6.jpeg]

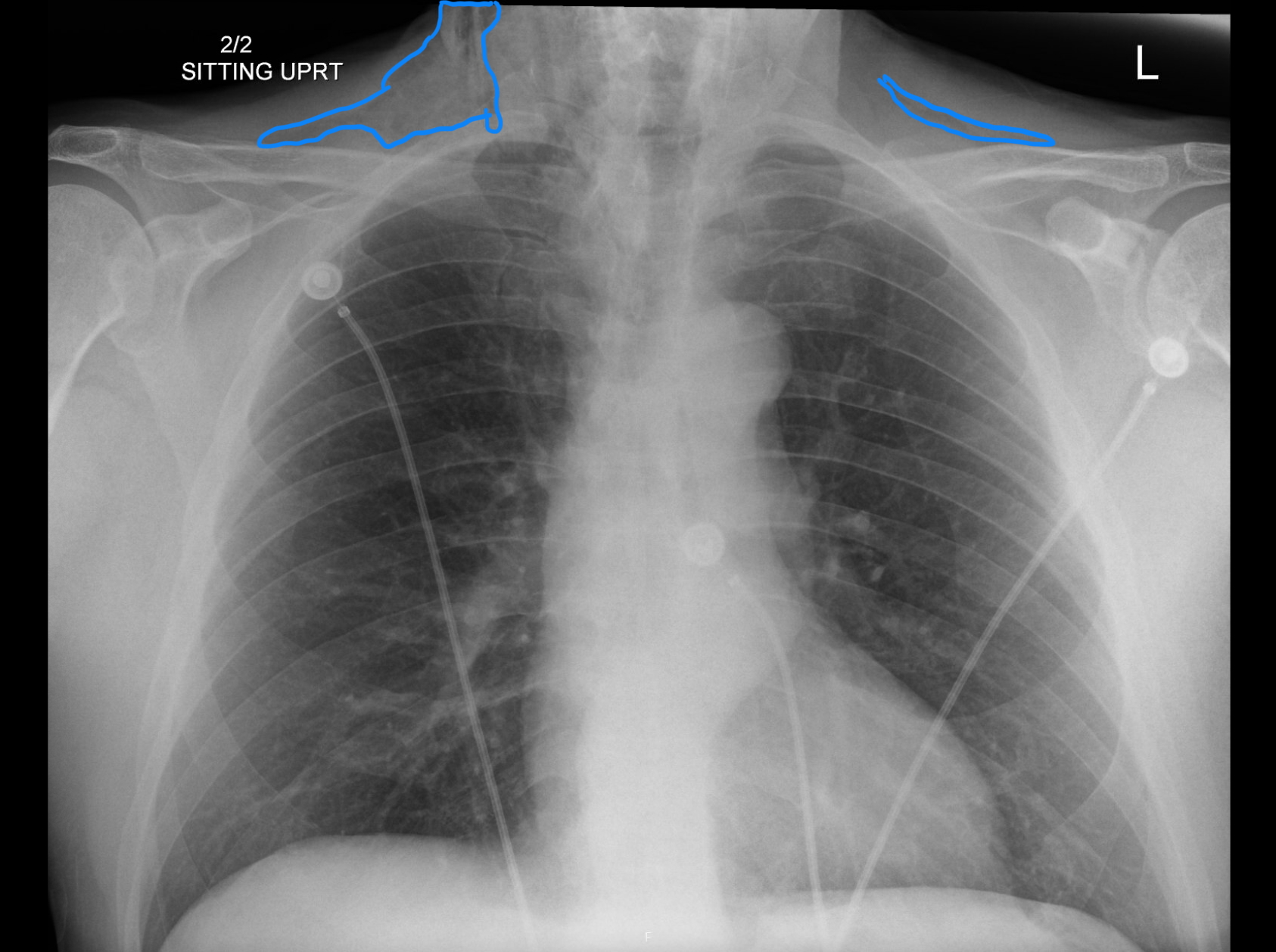

Supplement: Supplementary file 7 [file 10-3-V8-Supp7.jpeg]

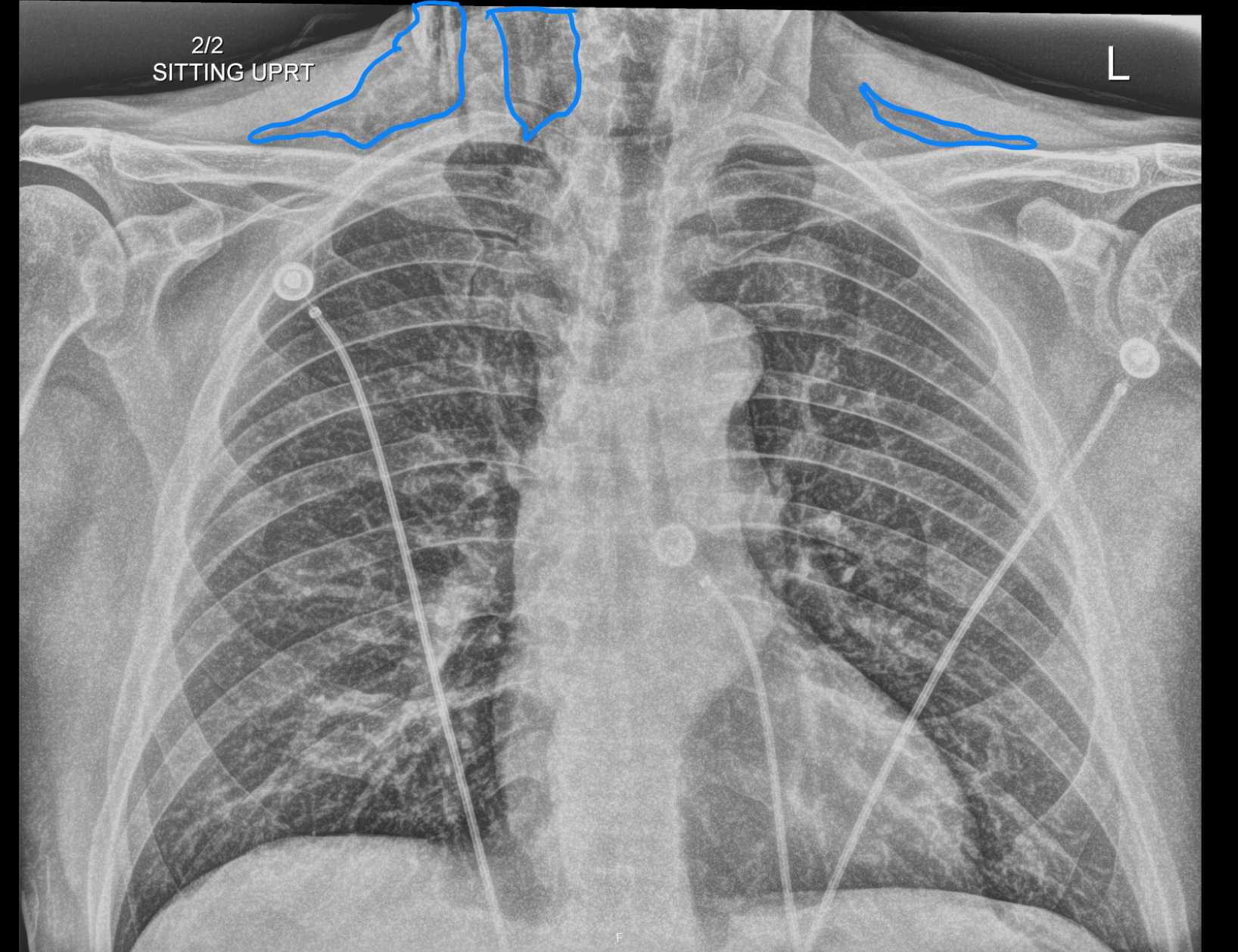

Supplement: Supplementary file 8 [file 10-3-V8-Supp8.jpeg]

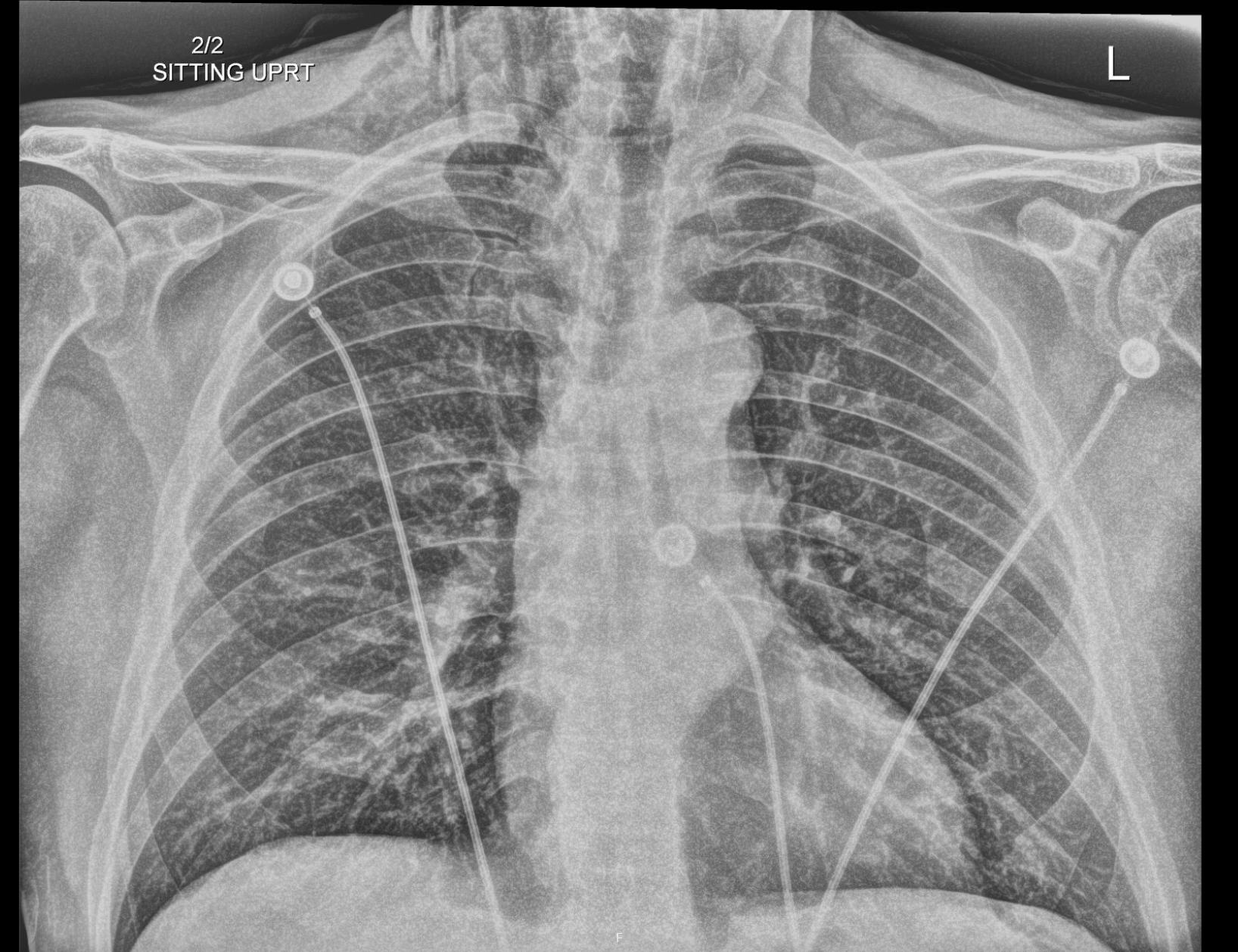

Supplement: Supplementary file 9 [file 10-3-V8-Supp9.jpeg]

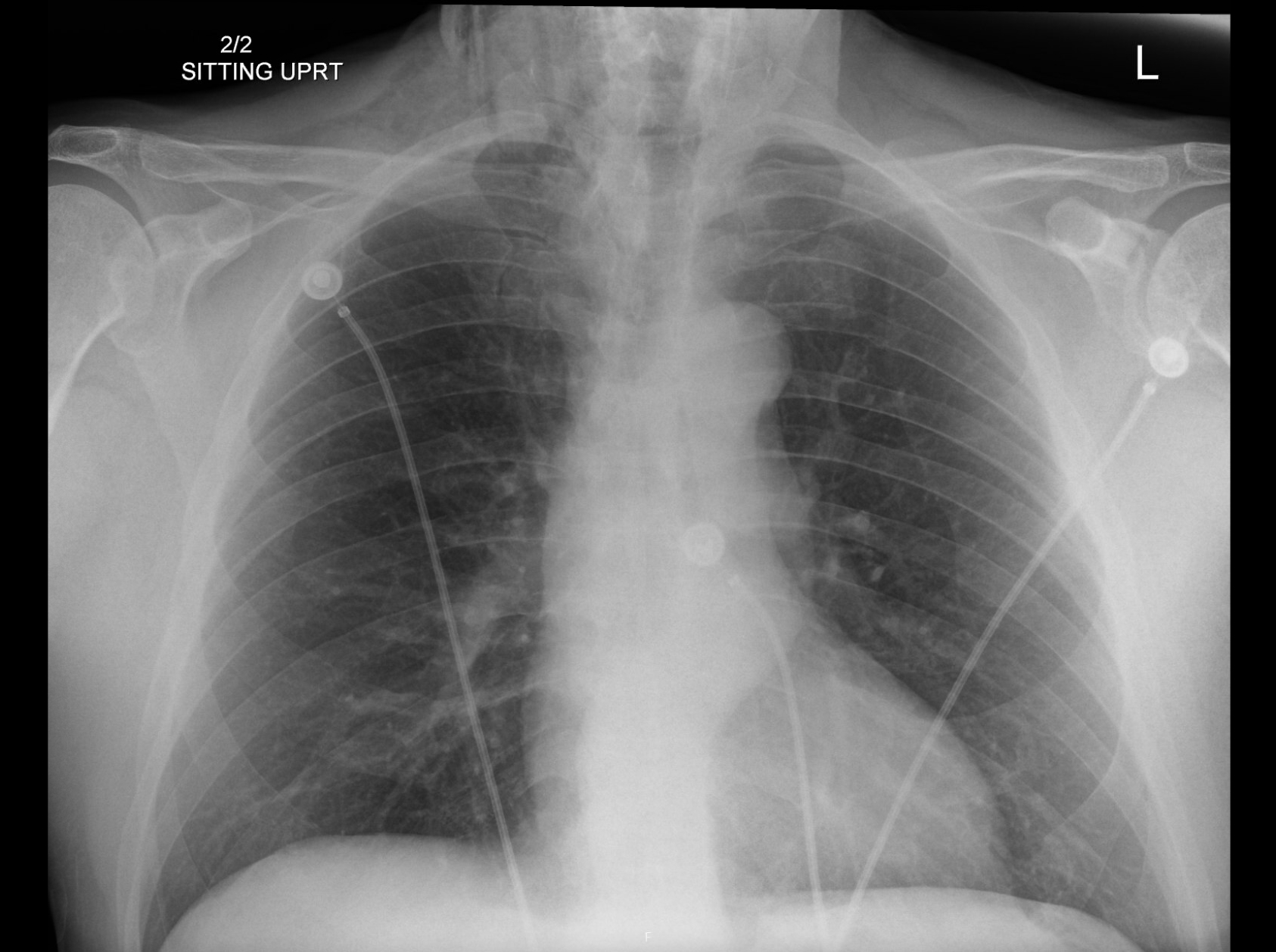

Supplement: Supplementary file 10 [file 10-3-V8-Supp10.jpeg]
